# Patterns of Hepatitis B, Hepatitis C and HIV Among Blood Donors in Samtah-Jazan Region

**DOI:** 10.1007/s44197-022-00051-7

**Published:** 2022-07-30

**Authors:** Abdullah A. Mobarki, Maymoon M. Madkhali, Gasim Dobie, Muhammad Saboor, Aymen M. Madkhali, Basem Madkhli, Yahia Hummadi, Abdullah Meshi, Hesham M. Al-Mekhlafi, Mohammad S. Akhter, Hassan A. Hamali

**Affiliations:** 1 Department of Medical Laboratory Technology, Faculty of Applied Medical Sciences, Jazan University, P.O. Box 1906, Gizan, 45142 Saudi Arabia; 2 Medical Research Center, Jazan University, Gizan, Saudi Arabia; 3 Samtah General Hospital, Jazan Health, Gizan, Saudi Arabia; 4 Translational and Clinical Research Institute, Faculty of Medical Sciences, Newcastle University, Newcastle, UK; 5 King Fahad Central Hospital, Jazan Health, Gizan, Saudi Arabia

**Keywords:** HBV, HCV, HIV, Transfusion-transmitted infections

## Abstract

**Background and Objective:**

Transfusion-transmitted infectious agents are amongst the major health burden worldwide. The purpose of this study was to evaluate the prevalence of hepatitis B virus (HBV), hepatitis C virus (HCV), and human immunodeficiency virus (HIV) among blood donors in Samtah General Hospital, Jazan region, Saudi Arabia.

**Material and Methods:**

In this retrospective study, blood donation records of all blood donors recruited between January 2019 and August 2020 were included for data acquisition. A total of 4977 blood donors’ records were reviewed and data were analysed.

**Results:**

Hepatitis B profile showed 0.60% blood donors positive for hepatis B surface antigen (HBsAg). Nucleic acid testing (NAT) showed the presence of HBV-DNA in 0.4% of the blood donors. Anti-HBs and anti-HBc antibodies were reactive in 3.34% and 7.31% blood donors’ units, respectively. Anti-HCV antibodies were reactive among 54 (1.09%) blood donors. Upon reviewing the NAT analysis results, 0.16% (08) blood donors showed the presence of HCV-RNA in their blood units. Anti-HIV antibodies were reactive in 8 (0.16%) blood donors.

**Conclusion:**

It is concluded that the frequency of HBsAg is comparatively lower while anti-HCV positivity is higher in Samtah, Jazan as a region compared to other regions of the country. Further studies are warranted to evaluate the cause of HCV infection in this area. Frequency of HIV is uncommon in this area.

## Introduction

Infectious agents transmitted through transfusion or sexually are amongst the major health burden worldwide. It is estimated that 2.3 million people die because of these infections annually [1]. Hepatitis B virus (HBV) and hepatitis C virus (HCV) are responsible for the deaths of 1.1 million individuals each year out of 3 million infected population [1]. HBV, HCV, HIV, *Plasmodium* species, and *Treponema pallidum* are the most common infectious agents associated with blood and/or blood components transfusion posing a major threat to the safety of blood [2]. Hence, strict donor recruiting criteria and screening for potential infections are essential to ensure the safety of blood and its components. The clinical history is evaluated, and high-risk blood donors are excluded from donation. The blood-borne viruses are screened through conventional serological methods and molecular techniques [3]. Various studies indicate that the implementation of such sensitive methods/techniques, which detect the infections early and precisely, have reduced the transmission of blood-borne pathogens through blood transfusion [4, 5]. Currently, serological markers for the evaluation of transfusion-transmitted infections (TTIs) include Hepatitis B surface antigen (HBsAg), anti-Hepatitis B core antibody (anti-HB core), anti-Hepatitis B surface antibodies (anti-HBs), anti-Hepatitis C virus (anti-HCV), anti-Human Immunodeficiency Virus antibodies (anti-HIV P24), anti-human T Lymphotropic Virus I/II antibodies (HTLV I/II), syphilis, and malaria. While molecular testing (by nucleic acid testing) of TTIs includes HBV-DNA, HCV-RNA and HIV-RNA [3].

The frequency of TTIs varies among blood donors geographically [5, 6]. Studies conducted in various parts of Saudi Arabia including Central [4, 7–10], Western [7, 11–13], and Eastern regions [14–16] have reported the prevalence of TTIs using serological techniques, NAT or both. While literature shows only one report from Jazan (southwestern region) indicating the prevalence of HBV and HCV among blood donors using serological techniques [17].

The availability of basic information regarding the frequency and distribution of TTIs among blood donors contributes to transfusion safety. Unfortunately, there is no data available describing the TTIs comprised of both serological and molecular methods among the blood donors from Jazan region. Therefore, this study aimed to evaluate the prevalence of HBV, HCV, and HIV among blood donors in Samtah City, Jazan region, southwestern Saudi Arabia.

## Material and Methods

### Study Settings

This retrospective study was conducted in Samtah General Hospital, Samtah, Jazan region. Prior to blood donation, all blood donors are evaluated following standard protocols and filling up a detailed unified proforma prepared by the Ministry of Health, Saudi Arabia. The proforma contains detailed demographic and questions related to clinical and infectious conditions which help in the selection of healthy blood donors. Prospective blood donors with a positive history of infectious or chronic disease are deferred from blood donation. Records of all blood donors recruited during January 2019 to August 2020 were included for data acquisition. A total of 4977 blood donors’ records were reviewed and data were extracted. Only blood bank and donation registries were reviewed.

### Donor Laboratory Testing

All the collected blood units were screened for TTIs in accordance with national donor screening guidelines. For serological testing, the automated chemiluminescent microparticle immunoassay analyzer ARCHITECT i2000 system (CMIA, Abbott Diagnostic, USA), with validated commercially available assays, was used for the detection of HBsAg (ARCHITECT HBsAg Qualitative), anti-HCV (ARCHITECT Anti-HCV), HIV p24 antigen and anti-HIV-1&2 (ARCHITECT HIV Ag/Ab Combo), anti-HBc (ARCHITECT Anti-HBc II), and anti-HBs (ARCHITECT Anti-HBs). All procedures were carried out according to the manufacturer's instruction, and the results were expressed as a signal to cut-off (S/CO), and S/CO > 1.0 was considered reactive. In addition to CMIA, blood units were also tested in parallel by individual nucleic acid test (ID-NAT) for HBV-DNA, HCV-RNA and HIV-1&2-RNA using The Procleix Ultrio Elite assay on the Panther system (Grifols, Spain) as indicated by the manufacturer's instructions. For initially reactive samples, the Ultrio Elite discriminatory assays for HIV-1/2, HCV, or HBV were performed.

### Ethical Considerations

Ethical approval was obtained from Jazan Health Ethics Committee, Ministry of Health, Saudi Arabia.

### Data Analysis

Donors’ data were entered in a Microsoft excel sheet; the collected data were analysed for the calculations for frequencies using Microsoft Excel software. Results were presented in frequency tables that show the distribution of studied diagnostic markers.

## Results

In the current study, data of 4977 blood donors who donated blood in Samtah General Hospital during January 2019 and August 2020 were reviewed and analysed. The current study comprises data of 3942 males representing 79.2% of the total reviewed blood donors, 27 female blood donors representing 0.5% and a group of 1008 (20.3%) blood donors whose gender was not cited. Mean age of the donors was 32.31 ± 7.8 (range 18–60 years). Approximately, 55.7% blood donors were Saudi, 23.6% were non-Saudi (other nationality) and the nationality of 20.8% was not mentioned. The majority of non-Saudi participants were Yemeni (16.8%; 841/4977) while 1.78% (89/4977) were Egyptian, 1.5% (75/4977)] Pakistani, 1.1% (55/4977) Indian, and less than 2.4% belonged to other nationalities.

Hepatitis B profile shows that 0.60% of the blood donors were positive for HBsAg. Nucleic acid testing showed the presence of HBV-DNA in 0.4% of the blood donors. Anti-HBs and anti-HBc antibodies were reactive in 3.34% and 7.31% blood donors unite, respectively (Table 1). In the studied population, anti-HCV antibodies were reactive among 54 (1.09%) blood donors (Table 1). Upon reviewing the NAT analysis results, 0.16% (*n* = 8) blood donors showed the presence of HVC-RNA in their blood units. Anti-HIV antibodies were reactive in 8 (0.16%) blood donors. Out of the eight blood units, one (0.02%) was confirmed positive through western blot analysis (Table 1). Table 2 shows further characterization of HBV profile markers in the studied population. A total of 29 (0.58%) blood units were not included, being blood units discarded due to either quantity not sufficient, hemolysis, leakage, or clotted, etc. Frequency of HCV and HIV according to nationality is shown in Table 3.

**Table 1:** Frequency of HBV, HCV and HIV tests results of the blood donors (*n* = 4977)

Markers	2019		2020		2019–2020	
	Negative *n* (%)	Positive *n* (%)	Negative *n* (%)	Positive *n* (%)	Negative *n* (%)	Positive *n* (%)
HBsAg	3098 (99.5)	15 (0.5)	1849 (99.2)	15 (0.8)	4947 (99.4)	30 (0.60)
HBV-DNA	3102 (99.6)	11 (0.4)	1856 (99.5)	9 (0.5)	4957 (99.5)	20 (0.40)
Anti-HBs	3001 (96.4)	112 (3.6)	1810 (97.1)	54 (2.9)	4811 (96.7)	166 (3.34)
Anti-HBc	2875 (92.4)	238 (7.6)	1738 (93.2)	126 (6.8)	4613 (92.7)	364 (7.31)
Anti-HCV	3077 (98.8)	36 (1.2)	1846 (99.0)	18 (1.0)	4923 (98.9)	54 (1.09)
HCV-RNA	3107 (99.8)	06 (0.2)	1862 (99.9)	02 (0.1)	4969 (99.8)	8 (0.16)
Anti-HIV	3109 (99.9)	04 (≅ 0.1)	1860 (99.8)	04 (0.2)	4969 (99.8)	8 (0.16)
HIV-RNA	3110 (99.9)	03 (≅ 0.1)	1862 (99.9)	02 (0.1)	4972 (99.9)	1 (0.02)

**Table 2:** Prevalence of HBV positive markers in the studied population (*n* = 4977)

	*N* Total population	% total population	Nationality		
			Saudi *n* = 2770	Non-Saudi *n* = 1174	Not mentioned n = 1033
HBsAg positive alone Anti HBc negative Anti HBs negative HBV DNA negative	6	0.12	5 (0.18%; male)	0	1 (0.1%)
HBsAg positive, Anti HBc positive and HBV-DNA positive	21	0.4	10 (0.36%; male)	Yemeni: 5 (0.42%)	6 (0.6%)
HBsAg negative and HBV-DNA positive	None				
HBsAg positive and anti HBc positive	3	0.46	3 (0.5%)	04	0
HBsAg negative and anti HBc positive	341	6.85	146 (5.27%)	Overall: 137 (11.67%) Afghani 3: (0.26%) Bangladeshi: 3 (0.26%) Egyptian: 2 (0.17%) Moroccan: 1 (0.09%) Indian: 4 (0.34%) Pakistani: 12 (1.0%) Philippines: 3 (0.26%) Sudanese: 10 (0.85%) Syrian: 4 (0.34%) Yemeni: 95 (8.1%)	58 (5.61%)
Anti HBc positive and anti HBs positive	166	3.33	70 (2.52%)	Overall: 70 (5.96%) Afghani: 3 (0.265%) Bangladeshi: 2 (0.17%) Egyptian: 2 (0.17%) Indian: 1 (0.09%) Pakistani: 5 (0.43%) Philippines: 2 (0.17%) Sudanese: 4 (0.34%) Syrian: 2 (0.17%) Yemeni: 49 (4.1)	26 (2.51%)
HBsAg negative and anti HBs positive	166	3.33	70 (2.52%)	Overall: 70 (5.96%) Afghani: 3 (0.26%) Bangladeshi: 2 (0.17%) Egyptian: 2 (0.17%) Indian: 1 (0.09%) Pakistani: 5 (0.43%) Philippines: 2 (0.17%) Sudanese: 4 (0.34%) Syrian: 2 (0.17%) Yemeni: 49 (4.1%)	26 (2.51%)
HBsAg positive and anti HBs positive	Nil				
All markers positive	Nil

**Table 3:** Characterization of positive HCV and HIV markers of blood donors according to nationality (*n* = 4977)

Reactive	Number (*n*)	Frequency (%)	Nationality	*n*	%
Anti-HCV	54	1.09	Saudi	26	0.5
			Yemeni	10	0.2
			Indian	1	0.02
			Pakistani	1	0.02
			Turkish	1	0.02
			Not mentioned	15	0.3
HCV-RNA	8	0.16	Saudi	4	0.08
			Yemeni	1	0.02
			Not mentioned	3	0.06
Anti-HIV	8	0.16	Saudi	4	0.08
			Yemeni	1	0.02
			Not mentioned	3	0.06
HIV-RNA	1	0.02	Not mentioned	1	0.02

## Discussion

Viral hepatitis is a leading cause of morbidity and mortality worldwide with huge health and economic burdens on countries [18]. In 2015, the world health organization (WHO) reported 1.34 million deaths due to viral hepatitis. Thus, the main aim of the Global Health Sector Strategy (GHSS) is to eliminate viral hepatitis by 2030 i.e., 90% reduction in new infections and 65% reduction in mortality [19]. In 2019, WHO estimated that 296 million individuals across the globe had chronic HBV infection. In addition, 1.5 million new infections and 920,000 deaths due to HBV occur annually [20]. HCV is also a prevalent infection infecting 130–185 million individuals worldwide [21, 22], with 1.75 million new infections in 2015 [23]. In Saudi Arabia, the prevalence of HBV was found to be 1.3–3.2% in 2019 [24], which was reduced from 8.3% reported in 1998 [25], while HCV positive ratio is up to 1.1% [9]. HIV is a known viral infection with deleterious effects with approximately 36 million cases in the world [26]. It is estimated that 3 out of 100,000 individuals are infected with HIV in the Saudi population [27].

This study reports the prevalence of TTIs including HBV, HCV, and HIV from Samtah General Hospital during 2019 and 2020. Of the 4977 donors’ blood donation records, overall, 580 had at least one positive marker of hepatitis B profile. HBsAg was tested positive in 0.60% of the studied population. Anti-HBs antibodies were detected in 3.34% of the blood donors. Out of 30 positive HBsAg blood donors, 20 (0.40%) had NAT positive results indicating infectious stage of the disease. In the current study, all HBsAg negative blood donors had negative HBV-DNA as well which excludes the presence of occult infection. Occult HBV infection in blood donors has also been reported in the literature which is contrary to the findings of the current study [28–30]. Non existence of HBsAg does not entirely eliminate the existence of the virus and there is still likelihood of HBV spreading from HBsAg negative donors through transfusion [16]. NAT positive results along with anti-HBc highlights the use of both these markers to eliminate any potential risk of HBV transmission thorough blood transfusion. The importance of NAT utilization in donor screening has been authenticated by several studies [16, 31]. This study shows that the results of positive HBV, within 2 years period, did not show any difference. Among the study participants, 3.34% showed their immunized status due to positive anti-HBs, while a total of 7.31% had positive anti-HBc.

It is important to note that the prevalence of HBsAg reported from Jazan Area in 2013 [17] is much higher (3.8%) than the current study (0.60%). The reason for these decreased rates could be attributed to the improvement plan and strategic action to eliminate HBV as reported previously [24]. Frequency of HBsAg in the current study shows similarities with some while differences with other published reports in Saudi Arabia as shown in Table 4. It is evident that frequency was higher in Tabuk, Riyadh, Aseer and Hail as compared to the current study [29, 32–34].

**Table 4:** Frequency of HBV serological markers reported in literature in Saudi Arabia (chronological order)

Study site	Year	Sample size	HBsAg	HBV-DNA	Anti-HBs	Anti-HBc	References
Riyadh	2000 –2002	24,173	1.5	NA	NA	NA	[8]
Jazan	2004–2009	29,949	3.8	NA	NA	5.7	[17]
Tabuk	2005–2006	3192	3.0	NA	NA	18.7	[32]
Riyadh	2011–2012	8501	0.7	8.6*	NA	2.3**	[29]
Makkah	2011–2014	22,963	0.7	0.72	NA	6.7	[13]
Dammam	2011–2015	22,842	0.28	0.05	0.05	2.91	[16]
Aseer Region	2012–2013	6679	1.03	0.96	NA	6.14	[36]
Hail	2013–2015	11,162	1.2	NA	NA	NA	[33]
Hail	2014–2015	361	8.6	NA	8.6	8.6	[34]
Majmaah	2015–2017	3028	0.33	0.46	7.80	9.81	[4]
Al Majmaah	2015–2017	3014	0.27	NA	NA	6.9	[31]
Riyadh	2016–2018	38,621	0.29	0.2	NA	4.0	[37]
Buraidah/Qassim	2017–2018	4590	0.08	NA	NA	0.78	[10]
Al Baha	2014 – 2017	3461	0.30	0.40	NA	7.3***	[28]
Samtah	2019 – 2020	4977	0.60	0.40	3.33	7.31	Current study

*Analysis of HBV-DNA 17 out of 198 samples (8.6%) yielded positive results and all of them were anti HBs-negative

**2.3% positive for anti HBc and these patients were suspected to have OBIs

***In absence of HBs Ag

Anti-HCV antibodies were detected in 1.09% of blood donors while NAT results showed that 0.16% of blood donors had HCV-RNA. The high rate of serological positivity for anti-HCV could be attributed to past infection while the HVC-RNA detection shows that 0.16% of blood donors have active HCV infection. Among the positive HCV blood donors, 0.58% were Saudi blood donors. Frequency of HCV in Yemeni blood donors was 0.22%, i.e., second highest. The overall prevalence of anti-HCV positive results is much higher in this area as compared to other parts of Saudi Arabia except Hail as shown in Table 5. This is an alarming situation that needs special attention to address. Further studies are required to find the route of transmission and epidemiology of HCV in this area.

**Table 5:** Comparison of the prevalence of HCV and HIV reported in different studies in Saudi Arabia

City /Region	Study period	Sample size (*n*)	Anti-HCV (%)	HCV-RNA (%)	Anti-HIV (%)	HIV-RNA (%)	References
Riyadh	2000 –2002	24,173	0.4	NA	0.0	NA	[8]
Jazan	2004–2009	29,949	0.41	NA	NA	NA	[17]
Makkah	2011–2014	22,963	0.44	0.05	0.07	0.03	[13]
Hail	2013–2015	11,162	0.04	NA	0.0	NA	[33]
Hail	2014–2015	361	7.2	NA	4.7	NA	[34]
Majmaah	2015–2017	3028	0.40	0.66	0.13	0.07	[4]
Riyadh	2016–2018	38,621	0.3	0.01	0.005	0.007	[37]
Buraidah/Qassim	2017–2018	4590	0.08	NA	0.0	0.0	[10]
Al Baha	2014–2017	3461	0.2	0.18	NA	NA	[28]
Samtah	2019–2020	4977	1.09	0.16	0.16	0.02	Current study

Evaluation of the anti-HIV and HIV-RNA results showed that 0.16% and 0.02%% blood donors were positive for both makers respectively. The frequency of anti-HIV positive donors is comparable with that of Makkah and Majmaah while lower than Riyadh. As apparent from Table 5, several studies did not find any positive case of anti-HIV and HIV-RNA. Low frequency of HIV could be due to several factors. Following Islamic principles and teachings that implies monogamous sexual relationship and prohibits adultery. Homosexuality, a leading cause of HIV and sexually transmitted infections in western countries, is not allowed in Islam [35]. Additionally, drug abusers face penalties if found guilty hence reducing the chances of intravenous drug abuse.

## Conclusion

It is concluded that the frequency of HBsAg in Samtah, Jazan region is comparatively lower than in other regions. The frequency of anti-HCV was high in the current study as compared to other studies. Further studies are warranted to evaluate the cause of HCV infection in this area. Prevalence of HIV in blood donors is least common in this area.

## Abbreviations

HBV: Hepatitis B virus
HCV: Hepatitis C virus
HIV: Human immunodeficiency virus
HBsAg: Hepatis B surface antigen
NAT: Nucleic acid testing
TTIs: Transfusion transmitted infections
anti-HB core: Anti-Hepatitis B core antibody
anti-HBs: Anti-Hepatitis B surface antibodies
anti-HCV: Anti-Hepatitis C virus
anti-HIV P24: Anti-Human Immunodeficiency Virus antibodies
HTLV I/II: Anti-human T Lymphotropic Virus I/II antibodies
CMIA: Chemiluminescent Microparticle Immunoassay
GHSS: Global Health Sector Strategy
WHO: World health Organization
